# Effect of LPS and IFN-γ transient inflammatory priming on periodontal ligament cells and Schwann cells

**DOI:** 10.1590/1807-3107bor-2026.vol40.079

**Published:** 2026-10-16

**Authors:** Luiza de Almeida Queiroz Ferreira, Natália Aparecida Gomes, Talyta Couto Freitas, Weihao Yuan, Adriely Cristina Costa Pinto, Lucca Sicilia, Ranulfo Benedito de Paula Miranda, Rodrigo Richard da Silveira, Alireza Moshaverinia, Mila Fernandes Moreira Madeira, Ivana Márcia Alves Diniz

**Affiliations:** (a) Universidade Federal de Minas Gerais, School of Dentistry, Department of Restorative Dentistry, Belo Horizonte, MG, Brazil.; (b) University of California, School of Dentistry, Section of Prosthodontics, Weintraub Center for Reconstructive Biotechnology, Los Angeles, CA, USA.; (c) Universidade Federal de Minas Gerais, Biological Sciences Institute, Department of Microbiology, Universidade Federal de Minas Gerais, Belo Horizonte, MG, Brazil.

**Keywords:** Periodontal ligament, Lipopolysaccharides, Schwann cells, Inflammation, In vitro techniques

## Abstract

This study investigated the effects of *Escherichia coli* lipopolysaccharide (LPS; 2,000 ng/mL) and interferon-γ (IFN-γ; 50 ng/mL) on human periodontal ligament cells (PDLCs) and mouse Schwann cells (SCs). After a transient inflammatory priming (5 hours), cell migration/proliferation and viability (n = 4) were assessed with scratch and MTT assays, respectively, while inflammatory markers were quantified by real-time quantitative polymerase chain reaction (RT-qPCR; n = 3). Mineralized matrix formation was induced for 12 days and analyzed via Alizarin Red staining (ARS; n = 4) and osteogenesis-related gene expression (RT-qPCR; n = 3). Because of their distinct origins, the two cell lineages were assessed independently. Exposure to LPS+IFN-γ did not affect the viability or migration/proliferation of PDLCs or SCs. In PDLCs, LPS+IFN-γ exposure upregulated interleukin-1α (IL-1α) and interleukin-6 (IL-6), interferon-γ (IFN-γ), IFN-γ receptor (IFN-γR), and tumor necrosis factor (TNF). In SCs, IL-6 and IFN-γR were upregulated, whereas TNF was downregulated compared to the untreated control. Following mineral matrix induction, exposed PDLCs showed increased expression of alkaline phosphatase (ALPL) and runt-related transcription factor 2 (RUNX-2). SCs upregulated ALPL, RUNX-2, and osteocalcin (OCN). Quantitative ARS data indicated that calcium deposition in PDLCs exposed to LPS+IFN-γ was similar to that in the untreated group. SCs exposed to LPS+IFN-γ exhibited significant mineralized matrix formation. Overall, PDLCs responded to LPS+IFN-γ priming with an inflammatory transcriptional response. SCs were unresponsive or showed a downregulated response to inflammatory markers (TNF and IL-1α). Both cell lineages demonstrated mineralized matrix formation after LPS and IFN-γ transient priming.

## Introduction

Periodontitis is a chronic inflammatory condition triggered by a shift from a symbiotic to a dysbiotic microbial community.^1^ It affects over 60% of adults worldwide, with nearly 24% presenting with a severe form.^2^ The disease is driven by a dysregulated host response, leading to connective tissue damage, irreversible alveolar bone loss, and a systemic inflammatory burden.^1^ Conventional clinical treatments, including surgical and nonsurgical procedures and antibiotic-based approaches, effectively control disease progression but have limited ability to reverse alveolar bone loss.^3^ To overcome these limitations, regenerative strategies that combine stem cells with biomaterials have emerged as promising approaches for restoring the PDL and alveolar bone.^4,5^ Successful tooth preservation is critical for reducing health inequalities and maintaining quality of life.^6^ However, the success of regenerative therapies depends on how transplanted cells respond to the inflammatory microenvironment.

During the course of periodontal disease, bacterial products such as LPS penetrate periodontal tissues and activate the host innate immune response, stimulating cytokine production.^7^ Identifying key cellular players that modulate the periodontal disease (PD) milieu is therefore essential for regenerative dentistry. Schwann cells (SCs) and their progenitors have emerged as promising candidates for tissue engineering due to their plasticity and secretome profile.^8–10^ Originating from the neural crest, these cells are required for peripheral nerve myelination and also serve as precursors during odontogenesis,^11^ giving rise to dental pulp cells, odontoblasts, and osteoblasts.^12^ Beyond their developmental roles, SCs orchestrate repair by secreting neurotrophic and growth factors, such as Platelet-derived growth factor AA (PDGF-AA) and Oncostatin M (OSM), thereby promoting the renewal of mesenchymal progenitor cells.^8^

The therapeutic potential of SCs is well documented in several tissues, including the skin.^8–10^ In the alveolar bone, SCs may promote tissue repair through paracrine signaling.^13,14^ Furthermore, their ability to modulate immune responses makes them promising candidates for the treatment of inflammatory diseases.^15^ Given that the periodontal ligament (PDL) is derived from the neural crest-derived ectomesenchyme of the dental follicle,^16^ SCs within the PDL and alveolar bone may promote homeostasis.^17^ However, how SCs respond to a chronic pro-inflammatory microenvironment remains unclear. The extent to which pathogen byproducts, such as LPS, influence SCs’ pro-inflammatory response and mineral matrix formation is underexplored. Moreover, for other cell types, such as periodontal ligament cells (PDLCs), evidence regarding their response to LPS remains conflicting.^18–20^ Therefore, we aimed to investigate the inflammatory and mineralized matrix formation responses of human PDLCs and mouse SCs following exposure to LPS from *Escherichia coli* and interferon-gamma (IFN-γ), as a transient inflammatory priming.

## Methodology

### Cell lineages and culture

PDLCs were isolated from PDL tissue from healthy patients according to previously published protocols.^5,21^ The tissue was digested in a solution containing 3 mg/mL type I collagenase and 4 mg/mL dispase for 1 hour at 37°C. PDL samples and single cells were obtained by passing the suspensions through a 70-μm cell strainer. The cells were seeded in Petri culture dishes, expanded in Dulbecco's Modified Eagle Medium (DMEM) supplemented with 15% fetal bovine serum, 1% penicillin, and 1% streptomycin (all from Thermo Fisher Scientific, Waltham, USA), and incubated at 37°C in 5% CO_2_. PDLC characterization was performed as previously described^23^. Briefly, PDLCs were incubated with CD105, CD146, CD45, and IgG antibodies and then subjected to flow cytometry, as described in previous reports.^5,21^ All procedures were approved by the Institutional Review Board of the University of California, Los Angeles (UCLA) under IRB#18-001947, and PDLCs between passages 4 and 8 were used for all experiments. A commercially available mouse SC line (ATCC-SW-10) was used for experimentation and expanded in the regular cell culture medium described above.

### LPS+ IFN-γ priming media

LPS+IFN-γ priming medium consisted of DMEM supplemented with 15% fetal bovine serum, 1% penicillin, and 1% streptomycin, supplemented with 2,000 ng/mL of LPS from *Escherichia coli* (Sigma Aldrich, Burlington, USA) and 50 ng/mL interferon gamma (IFN-γ; Sigma Aldrich, Burlington, USA) for 5 hours.^22^

### Migration/proliferation and viability

PDLCs and SCs were seeded in quadruplicate for each group at a density of 2 x 10^4^ cells/well in a 48-well plate. After reaching confluence, a scratch was created at the center of each well using a P200 pipette tip in both untreated and LPS+IFN-γ-treated cells. Bright-field images were obtained after scratching (0 h) using a Leica DMi8 microscope (Wetzlar, Germany), after which the cells were cultured in regular DMEM. Additional images were obtained after 24 and 48 hours to assess cell migration/proliferation. Wound areas were quantified using ImageJ (NIH, Bethesda, USA). The software was calibrated using the microscope scale bar, and the wound area was outlined at all time points by a single calibrated examiner using the freehand selection tool. The percentage of wound closure was calculated as follows: (initial wound area – wound area at 24 or 48 h) / initial area ×100.

At 96 hours, cell viability was assessed using the MTT assay (Sigma-Aldrich, St. Louis, USA) according to the manufacturer's instructions. Absorbance was read at 540 nm using a spectrophotometer (BioTek Synergy H1, BioTek Instruments, Winooski, USA).

### Inflammatory assessment in a microenvironment containing LPS and IFN-γ

PDLCs and SCs were seeded in triplicate for each group in 12-well plates at a density of 1 × 10^5^ cells/well. Upon reaching confluence, the LPS+IFN-γ group was exposed. At 24 hours, SCs or PDLCs were collected in TRIzol (Invitrogen, Waltham, USA) for real-time quantitative polymerase chain reaction (RT-qPCR) of pro-inflammatory markers: interleukin-1-alpha (*IL1-*μ), interleukin-6 (*IL-6*), tumor necrosis factor (*TNF*), *IFN-*γ, interferon-gamma receptor (*IFN-*γ*R*). Primer sequences for human (PDLCs) or mouse (SCs) were obtained from PrimerBank (https://pga.mgh.harvard.edu/primerbank/) and synthesized by Thermo Fisher (Thermo Fisher Scientific, Waltham, USA). Primer sequences are described in Table 1.

**Table 1:** Sequence of primers used for pro-inflammatory gene expression with q-PCR.

Gene	Primers
*mIL6*	*Forward:* TAGTCCTTCCTACCCCAATTTCC
	*Reverse:* TTGGTCCTTAGCCACTCCTTC
*hIL6*	*Forward:* ACTCACCTCTTCAGAACGAATTG
	*Reverse:* CCATCTTTGGAAGGTTCAGGTTG
*mIL1*a	*Forward:* CGAAGACTACAGTTCTGCCATT
	*Reverse:* GACGTTTCAGAGGTTCTCAGAG
*hIL1*a	*Forward:* TGGTAGTAGCAACCAACGGGA
	*Reverse:* ACTTTGATTGAGGGCGTCATTC
*mIfng*	*Forward:* ATGAACGCTACACACTGCATC
	*Reverse:* CCATCCTTTTGCCAGTTCCTC
*hIfng*	*Forward:* TCGGTAACTGACTTGAATGTCCA
	*Reverse:* TCGCTTCCCTGTTTTAGCTGC
*mIfngr*	*Forward:* CTGGCAGGATGATTCTGCTGG
	*Reverse:* GCATACGACAGGGTTCAAGTTAT
*hIfngr*	*Forward:* TCTTTGGGTCAGAGTTAAAGCCA
	*Reverse:* TTCCATCTCGGCATACAGCAA
*hTNF*	*Forward: GAGGCCAAGCCCTGGTATG*
	*Reverse: CGGGCCGATTGATCTCAGC*
*mTNF*	*Forward: CCCTCACACTCAGATCATCTTCT*
	*Reverse: GCTACGACGTGGGCTACAG*
*mGapdh*	*Forward:* AGGTCGGTGTGAACGGATTTG
	*Reverse:* TGTAGACCATGTAGTTGAGGTCA
*hGapdh*	*Forward:* GGAGCGAGATCCCTCCAAAAT
	*Reverse:* GGCTGTTGTCATACTTCTCATGG

Total mRNA was extracted using the PureLink RNA kit (Thermo Fisher Scientific, Waltham, MA, USA) following the TRIzol protocol. Subsequently, 0.37 μg of total RNA was reverse transcribed using the qScript cDNA kit (Quantabio, Beverly, USA). The obtained cDNA was used for qPCR with SYBR Green PCR Master Mix (Thermo Fisher Scientific, Waltham, USA). Ct values for pro-inflammatory markers, obtained in triplicate, were normalized to the endogenous control, glyceraldehyde-3-phosphate-dehydrogenase (*GAPDH*) and compared with the reference control group (untreated with LPS+IFN-γ) for analysis of inflammatory gene expression using the 2^−ΔΔCt^ method.

### Mineral matrix formation

For the calcium deposition assay, PDLCs and SCs were seeded in triplicate onto 12-well plates at a density of 1 × 10^5^ cells/well for each experimental group. Cells were cultured in mineralization induction medium (DMEM supplemented with 15% FBS, 1% penicillin, and streptomycin, 0.1 mM L-ascorbic acid, 0.01 μM dexamethasone, 1.8 mM KH_2_PO_4_). Cells cultured in regular conditions were also evaluated. The medium was changed every 48 hours.

After 12 days, calcium deposition nodules and mineralized matrix were evaluated by Alizarin Red staining (ARS). Cells were washed with PBS, fixed in 75% isopropanol, rehydrated with Milli-Q water (Merck, Darmstadt, Germany), and incubated with ARS solution (pH 7.2) for 5 minutes. After washing with PBS and air-drying, bright-field images were acquired using a Leica DMi8 microscope (Wetzlar, Germany). Mineral deposits were dissolved in a 10% acetic acid-methanol solution (4:1, v/v). Absorbance was measured using a spectrophotometer at 490 nm (BioTek Synergy H1, BioTek Instruments, Winooski, VT, USA). The values were normalized to the total protein absorbance of each well.

Cells were also collected for RT-qPCR analysis of osteogenesis-related genes, including alkaline phosphatase (*ALPL*), runt-related transcription factor 2 (*RUNX-2*), and osteocalcin (*OCN*), using the primer sequences listed in Table 2. RT-qPCR was conducted as described above. The untreated group cultured in regular media served as the reference control.

**Table 2:** Sequence of primers used for osteogenic gene expression with q-PCR.

Gene	Primers
*mRunx-2*	*Forward:* ATGCTTCATTCGCCTCACAAA
	*Reverse:* GCACTCACTGACTCGGTTGG
*hRunx-2*	*Forward:* TGGTTACTGTCATGGCGGGTA
	*Reverse:* TCTCAGATCGTTGAACCTTGCTA
*mAlpl*	*Forward:* CCAACTCTTTTGTGCCAGAGA
	*Reverse:* GGCTACATTGGTGTTGAGCTTTT
*hAlpl*	*Forward:* ACCACCACGAGAGTGAACCA
	*Reverse:* CGTTGTCTGAGTACCAGTCCC
*mOcn*	*Forward:* GGGTGCAGAACAGACAAGTCCCA
	*Reverse:* AGCTCCAAGGTAGCGCCGGA
*hOcn*	*Forward:* CACTCCTCGCCCTATTGGC
	*Reverse:* CCCTCCTGCTTGGACACAAAG
*mGapdh*	*Forward:* AGGTCGGTGTGAACGGATTTG
	*Reverse:* TGTAGACCATGTAGTTGAGGTCA
*hGapdh*	*Forward:* GGAGCGAGATCCCTCCAAAAT
	*Reverse:* GGCTGTTGTCATACTTCTCATGG

### Statistical analysis

Data were checked for normality using the Shapiro-Wilk test, and homogeneity of variances was assessed with Levene's test (p > 0.05). For comparison between two groups, normally distributed data were analyzed using an unpaired Student's *t*-test (*p* < 0.05). For multiple group comparisons across different time points and conditions, a repeated-measures two-way ANOVA followed by Tukey's post-hoc test was used. For comparison among multiple groups, a one-way ANOVA followed by Tukey's post-hoc test was used. Data are presented as mean ± standard error of the mean (SEM). Statistical analyses were performed using GraphPad Prism 9.0 (GraphPad Software, Version 9.0, La Jolla, USA). Statistical significance was set at *p* < 0.05 (95% confidence level). All experiments were analyzed using the number of biological replicates specified for each assay.

## Results

Because of the distinct origins of the PDLCs and SCs used in this study, the results are presented separately.

### Effect of LPS+IFN-γ priming on migration/proliferation and viability

LPS+IFN-γ exposure did not impair PDLC (1b) migration/proliferation, assessed as the percentage of remaining wound area at 24 and 48 hours (Figure 1a) (p > 0.05). The treatment numerically reduced SC migration/proliferation (1c), with no statistical difference compared with the control group (p > 0.05).

**Figure 1:**
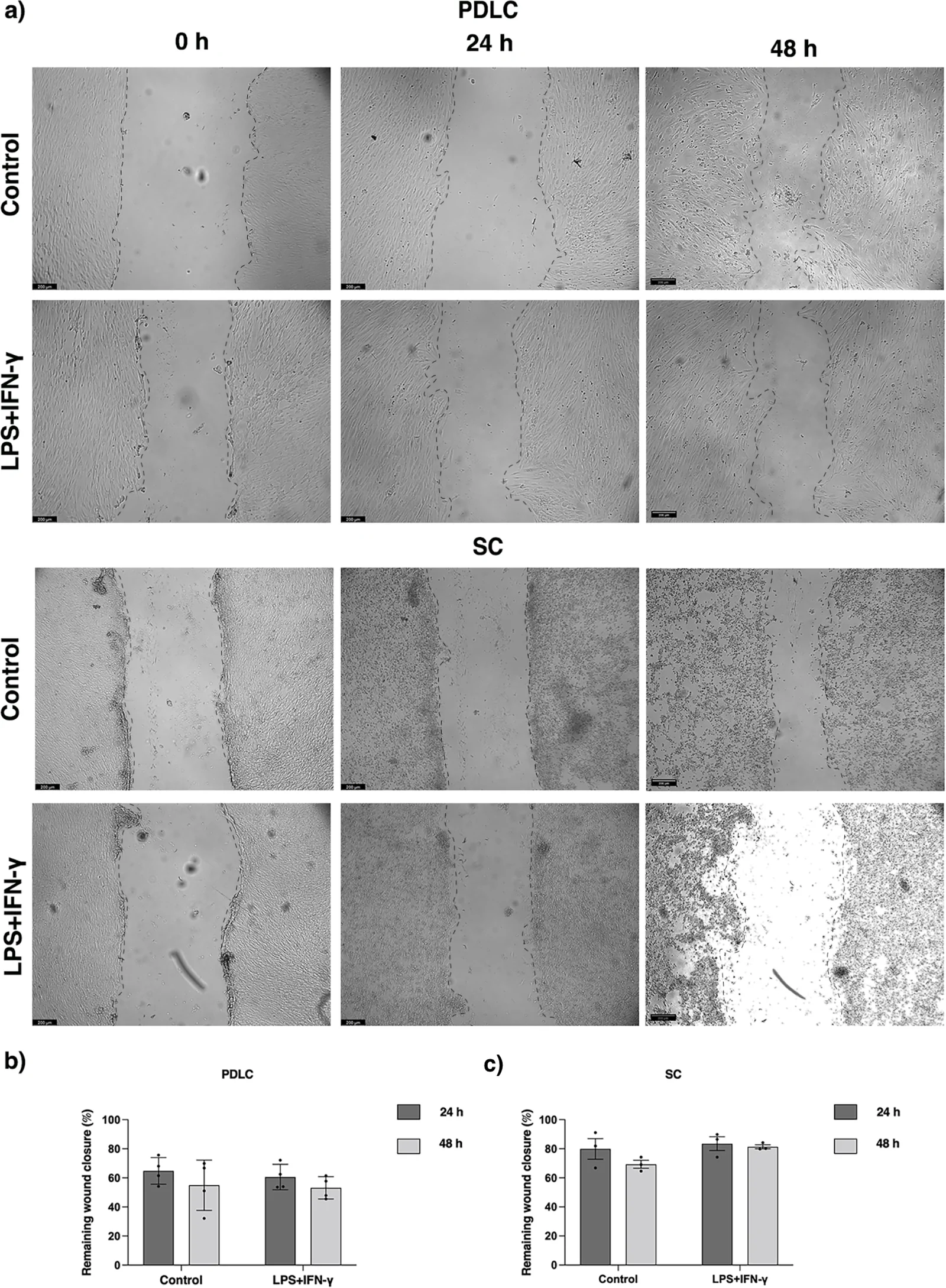
Migration/proliferation of PDLCs and SCs with or without LPS+IFN-γ.

PDLC: periodontal ligament cell; SC: Schwann cell; LPS: lipopolysaccharide; IFN-γ: interferon-γ. (a) Representative bright-field images of scratch assay at 0 h, 24 h, and 48 h. Scale bar = 200 μm; (b) Bar graph showing the percentage of remaining wound area for PDLCs with or without LPS+IFN-γ priming; (c) Bar graph showing the percentage of remaining wound area for SCs with or without LPS+IFN-γ priming. Data are expressed as mean ± standard error of the mean (SEM). Comparisons between groups and time points (two-way repeated measure ANOVA followed by Tukey's post-hoc test) showed no statistically significant differences (p > 0.05, n = 4).

At 96 hours, cell viability was unaffected by LPS+IFN-γ treatment (Figures 2a and b). The differences between the control and treated groups were not significant for any cell type analyzed (p > 0.05).

**Figure 2:**
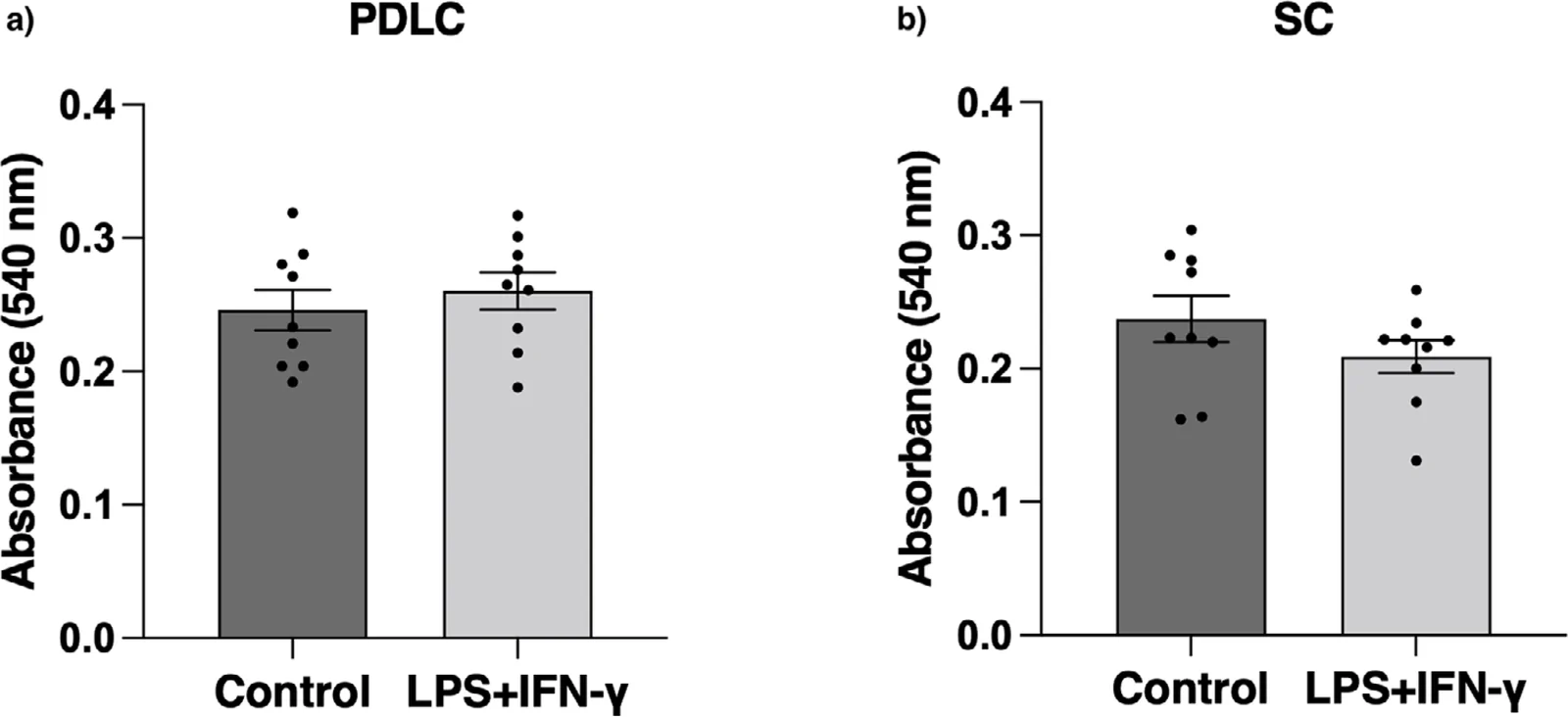
Viability of PDLCs and SCs with or without LPS+IFN-γ.

PDLC: periodontal ligament cell; SC: Schwann cell; LPS: lipopolysaccharide; IFN-γ: interferon-γ. (a) Bar graph showing the viability of PDLCs untreated or treated with LPS+IFN-γ; (b) Bar graph showing the viability of SCs untreated or treated with LPS+IFN-γ. Data are expressed as mean ± standard error of the mean (SEM). Comparisons between groups (control or LPS+IFN-γ) by *t*-test revealed no significant differences (p > 0.05, n = 4).

### Effect of LPS+IFN-γ priming on expression of pro-inflammatory markers

LPS+IFN-γ-treated PDLCs showed a significant upregulation of *IL-1* a, *IL-6*, *IFN-*γ, *IFN-*γ*R*, and *TNF* at 24 h compared to the untreated group (p < 0.05) (Figures 3a–e).

**Figure 3:**
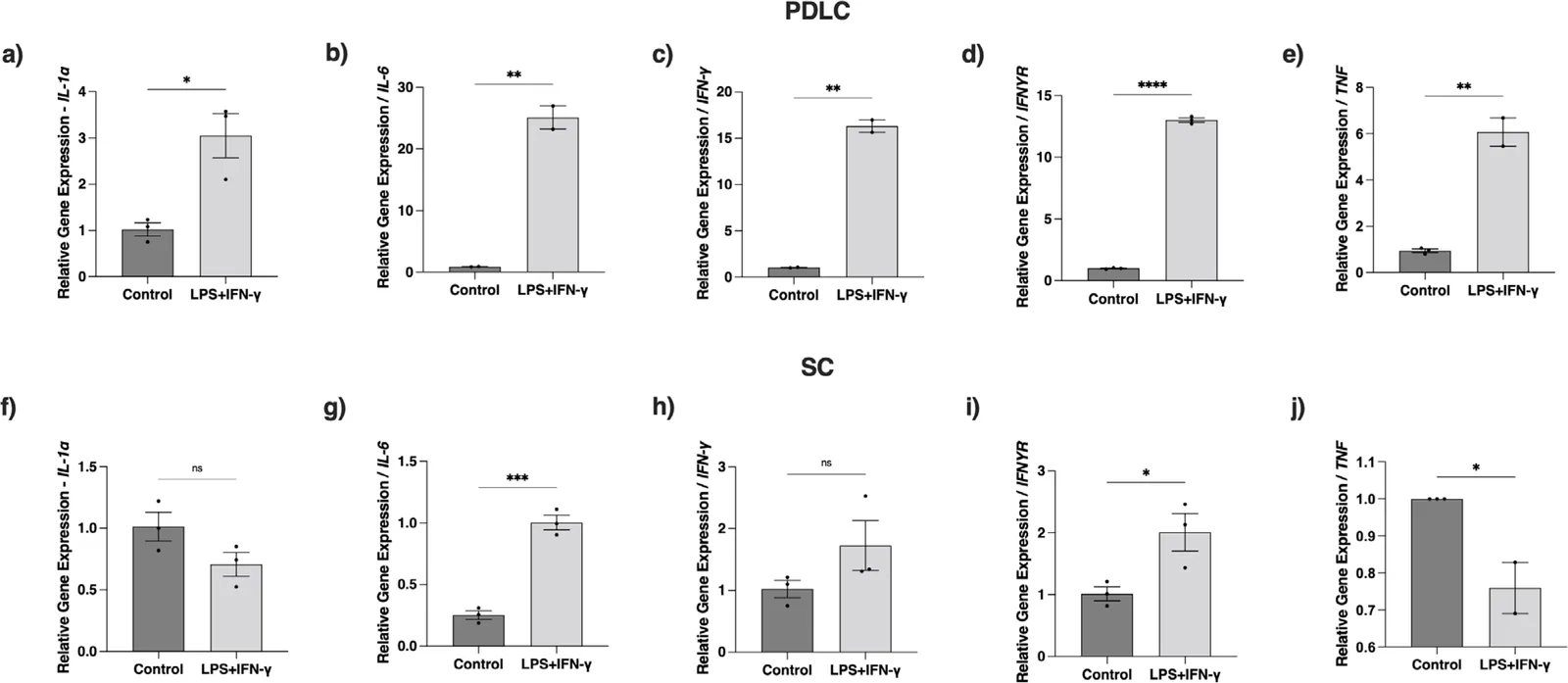
Expression of pro-inflammatory genes by PDLCs or SCs cultured within a microenvironment containing LPS+IFN-γ.

ns: non-significant; SC: Schwann cell; LPS: lipopolysaccharide; IFN-γ: interferon-γ; IL1α: interleukin 1-α; IL-6: interleukin 6; IFN-γ: interferon gamma; IFN-γR: interferon gamma receptor; TNF = tumor necrosis factor. Bar graphs showing relative gene expression of pro-inflammatory genes in PDLCs (a-e) and SCs (f-j) treated with or without LPS+IFN-γ. Data are expressed as mean ± standard error of the mean (SEM). Unpaired t-test. *p < 0.05; **p < 0.01; ***p < 0.0005; ****p < 0.0001; (n = 3). PDLC = periodontal ligament cell.

In SCs, *IL-1*a expression was downregulated (Figure 3f), and *IFN-*γ expression was slightly increased (Figure 3h), but without a significant difference when compared to the untreated group (p *>* 0.05). SCs showed significant upregulation of *IL-6* and *IFN-*γ*R*, while *TNF* expression was downregulated after LPS+IFN-γ exposure (p *<* 0.05) (Figures 3g, i, and j).

### Effect of LPS+IFN-γ priming on mineral matrix formation

When evaluating the ARS color bright-field images, PDLCs cultured in regular media showed minimal staining, whereas PDLCs cultured in mineral-induction media exhibited an increased mineralized matrix and nodule formation after LPS+IFN-γ priming (Figure 4a). Quantitative analysis showed that mineral matrix formation was similar between the untreated and LPS+IFN-γ groups of PDLCs (p>0.05) (Figure 4b).

**Figure 4:**
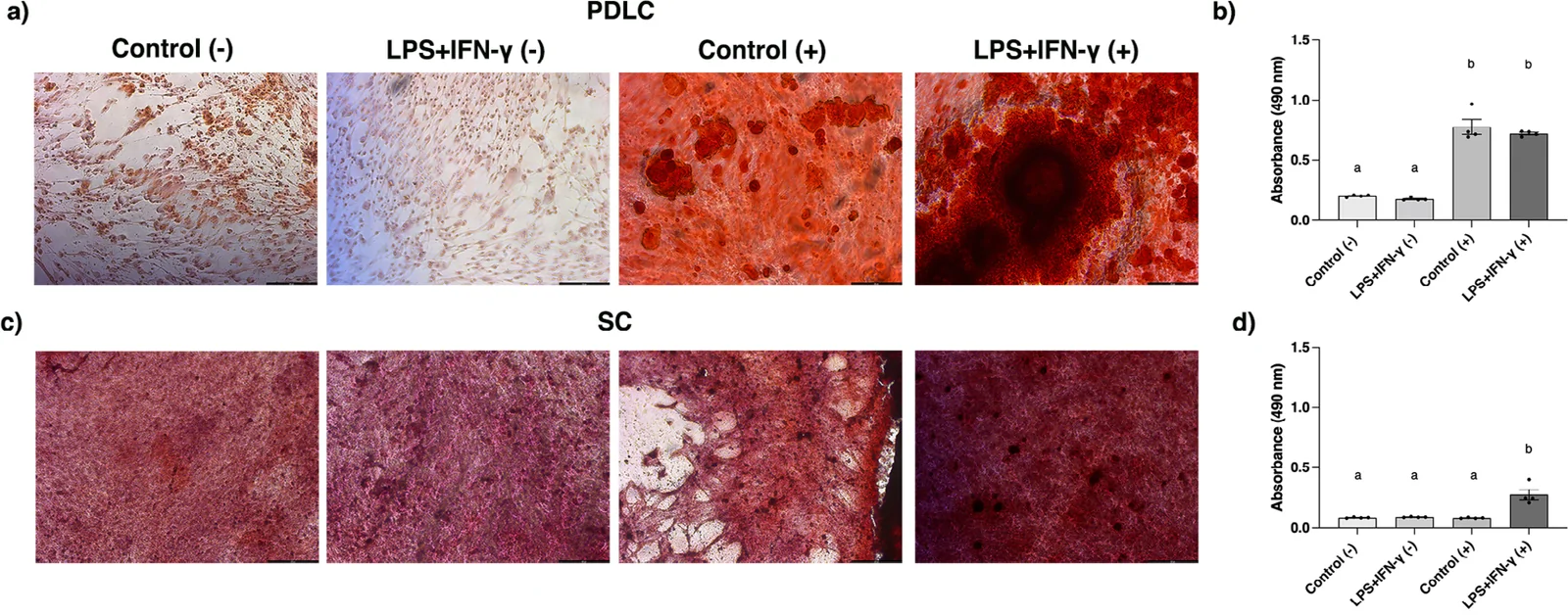
Alizarin Red staining bright-field images and quantification of PDLCs and SCs cultured in a microenvironment containing LPS+IFN-γ for 12 days.

PDLC: periodontal ligament cell; SC: Schwann cell; LPS: lipopolysaccharide; IFN-γ: interferon-γ; (-) = regular media; (+) = mineral-induction media. Representative bright-field images of Alizarin Red staining of PDLCs (a) and SCs (c). Bar graph showing the quantitative analysis in PDLCs (b) and SCs (d). One-way ANOVA followed by Tukey's multiple comparisons test. (n = 3). Data are expressed as mean ± standard error of the mean (SEM). Different letters indicate a significant difference among groups (p < 0.05).

For SCs, ARS staining revealed enhanced mineral deposition when cells were cultured in mineral-induction media and exposed to LPS + IFN-γ compared to the untreated control (Figure 4c and d). Groups cultured in regular media showed no significant mineral matrix formation.

### Effect of LPS+IFN-γ priming on osteogenesis-related gene expression

When evaluating the effect of LPS+IFN-γ on osteogenesis-related markers, PDLCs cultivated in mineral-induction media upregulated *ALPL* and *RUNX-2* (p < 0.05), while downregulated *OCN* (p < 0.05) (Figures 5a-c).

**Figure 5:**
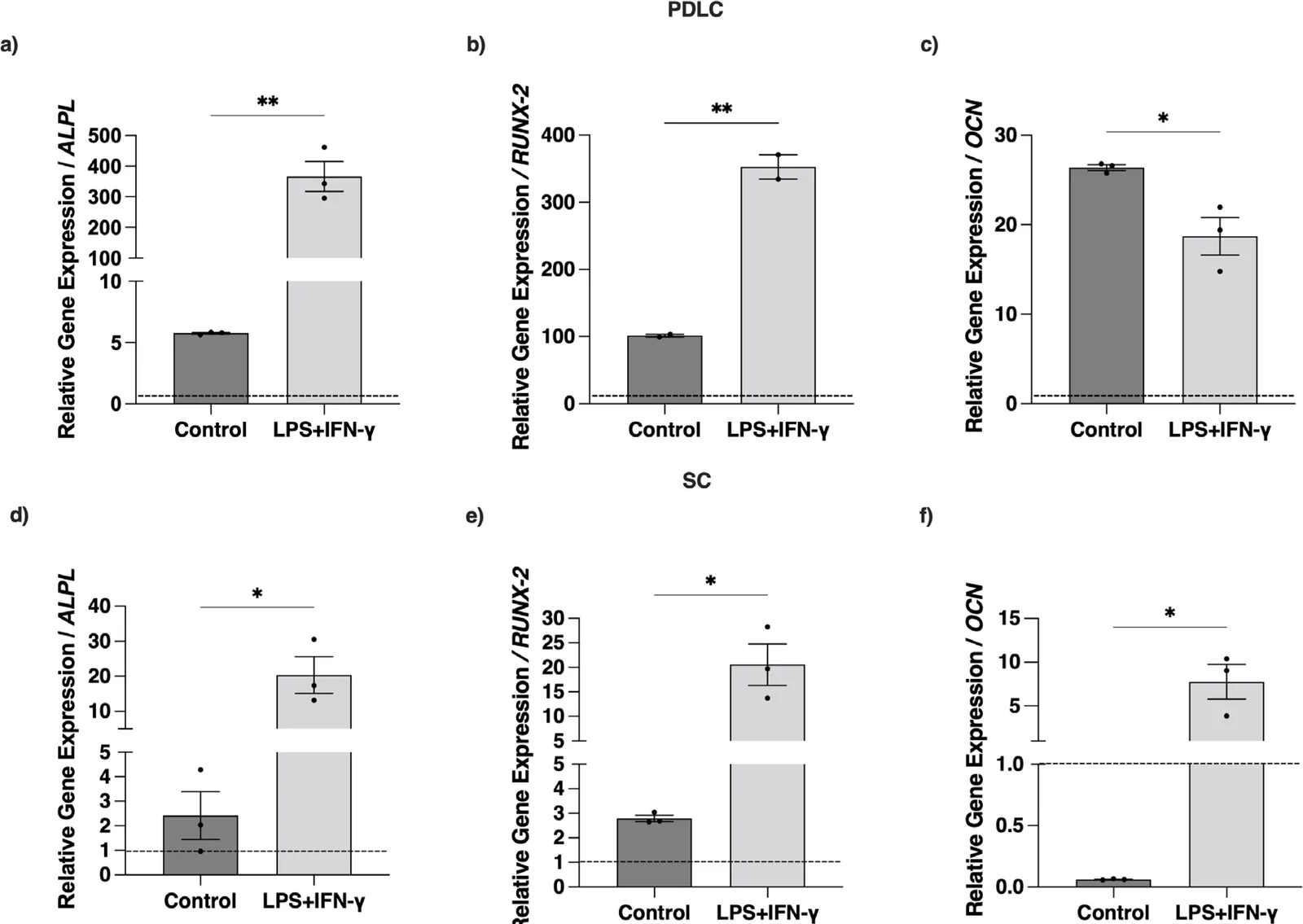
Expression of osteogenesis-related genes in PDLCs and SCs cultured in a microenvironment containing LPS+IFN-γ for 12 days.

PDLC: periodontal ligament cell; SC: Schwann cell; LPS: lipopolysaccharide; IFN-γ: interferon-γ; RUNX-2: RUNX family transcription factor 2; OCN: osteocalcin; ALP:= alkaline phosphatase. Bar graphs showing the relative gene expression of osteogenesis-related genes in PDLC (a-c) and SC (d-f) treated with or without LPS+IFN-γ. Data are expressed as mean ± standard error of the mean (SEM). Unpaired t-test. *p < 0.05; **p < 0.01. The dotted line indicate cells cultivated in regular media (n = 3).

SCs cultivated in mineral-induction media upregulated all assessed osteogenesis-related genes (*ALPL*, *RUNX-2,* and *OCN)* when exposed to LPS+IFN-γ (p < 0.05) (Figures 5d-f)

## Discussion

Upon injury, neural crest-derived stem cells respond by secreting growth factors and undergoing differentiation, which may help prevent tissue loss. Mechanisms such as increased blood supply and neural response are activated to promote tissue repair.^9^ In the present study, we evaluated the effect of LPS derived from *E. coli* and IFN-γ on PDLC and SC functions. The inflammatory priming consisted of a transient challenge, a standardized method for inducing a pro-inflammatory response.^22,23^ *E. coli* LPS enhances the expression of inflammatory cytokines and TLR4.^23^ Moreover, LPS in the presence of IFN-γ induces TNF production and upregulates CD14 receptors, thereby enhancing immune activation in monocytes and macrophages.^22^ This association increases inflammation through cytokine receptor expression, autocrine and paracrine signaling, and activation of downstream response genes,^22^ enabling the in vitro simulation of several inflammatory aspects of periodontal inflammation. Our data demonstrated that LPS+IFN-γ increased the inflammatory response in PDLCs but did not alter their mineral matrix formation capacity, viability, or migration/proliferation. In SCs, transient inflammatory priming elicited only a mild inflammatory response while enhancing mineral matrix formation, without compromising viability or migration/proliferation.

Cell proliferation and migration are essential for tissue repair.^24^ Our results demonstrated that PDLC and SC migration/proliferation and viability were not affected by the inflammatory microenvironment containing LPS at 2,000 ng/mL. Consistent with our findings for PDLCs, a previous study showed that the proliferation and viability of PDLCs were not affected by *E. coli* LPS at concentrations of 100 and 1,000 ng/mL immediately after treatment (0 h) and after 24, 48, or 72 hours. Moreover, the percentage of viable cells exposed to LPS was similar to that in the untreated group.^20^ Another study reported that *E. coli* LPS at higher concentrations (1,000 ng or 10,000 ng/mL) over 10 days did not affect PDLC proliferation.^18^ Likewise, a previous study reported no significant changes in SC viability when treated with LPS (10 μg/μL).^25^

During the progression of periodontal disease, LPS, a component of the outer membrane of gram-negative bacteria, triggers an inflammatory response in cells within or surrounding the affected tissue.^26,27^ Our data showed that PDLCs stimulated with LPS+IFN-γ upregulated *IL-1*β, *IL-6*, *IFN-*γ, *IFN-*γ*R*, and *TNF*. Other studies are consistent with this finding, as *E. coli* LPS induces a pro-inflammatory response in PDLCs, with increased expression of cytokines such as *IL-1*, *TNF*, *IL-6*, and *IL-8.* ^19,28^ Based on the current literature, this response is likely related to the physiological role of PDLCs gin the periodontal ligament, which primes these cells to respond to the inflammatory microenvironment by activating nuclear factor-kappa (NF-κβ) and MAPK pathways, and their sensitivity to TLRs.^29^

SCs primarily regulate neural response; however, they also participate in immune responses by recognizing and presenting antigens and secreting cytokines.^30^ Following nerve injury, SCs have been shown to produce pro-inflammatory cytokines such as IL-1β, IL-6, and TNF,^31^ through the activation of the NF-κβ pathway.^25^ In our study, SCs upregulated the expression of *IL-6*, *IFN-*γ, *IFN-*γ*R* and downregulated *IL1-*α and *TNF*. Although these results should be interpreted cautiously because they were obtained following a transient priming challenge, the downregulation of cytokines by SCs might reflect an attempt to control inflammation. SCs can modulate local immune responses through major histocompatibility complex class II (MHC-II) during inflammation^32^ and contribute to the restoration of neural homeostasis during tissue repair.^33,34^

Cytokines produced during inflammatory conditions such as periodontitis affect cell poptions, leading to either decreased or increased bone remodeling.^35^ We therefore analyzed the mineralized matrix formation and osteogenesis-related genes in PDLCs and SCs after LPS+IFN-γ transient inflammatory priming. We observed that PDLCs upregulated *ALPL* and *RUNX-2*. Previous data on mineralization properties of PDLCs when exposed to LPS are inconsistent.^19,20^ Downregulation of *RUNX-2*, *ALPL*, and *OCN*, along with decreased ARS staining indicating reduced mineralized matrix deposition, was reported when PDLCs were stimulated with *E. coli* LPS. These findings suggest that LPS-induced inflammation can alter the PDLC phenotype and affect their osteogenic differentiation capacity.^20^ Consistent with our data, another study showed mineralized matrix formation as detected by ARS at 21 days when periodontal ligament progenitor cells were stimulated with *E. coli* LPS, and reported upregulation of *RUNX-2*, *ALPL*, and *OCN* after seven days.^19^ In contrast, another study reported that stimulation of PDLCs with *Porphyromonas gingivalis* LPS had no effect on mineral matrix formation.^18^

All marker genes evaluated in this study were upregulated in SCs following LPS+IFN-γ priming. To our knowledge, no studies have examined the effects of LPS+IFN-γ or LPS alone on the osteogenic potential or mineralized matrix formation of SCs. SCs cultured with pre-osteoblasts exhibit increased mineralization activity, suggesting that they may contribute to bone repair.^13,14^ Further evidence shows that SC activation could play a role in periodontitis by modulating alveolar bone loss and inflammation.^36^ However, the mechanisms underlying SCs’ mineral matrix formation in response to inflammatory stimuli are largely underexplored and deserve further investigation.

This study has several limitations. First, it used *E. coli*-derived LPS instead of *P. gingivalis*-derived LPS. The choice of stimulus, whether a typical endotoxin, such as *E. coli* LPS, or to LPS from another gram-negative pathogen, such as *P. gingivalis*, can differentially affect the cellular response. LPS from *P. gingivalis* primarily activates Toll-like receptor 2 (TLR2), whereas LPS from *E. coli* is a well-established and reproducible agonist of TLR4-mediated signaling pathways. Second, although PDLCs were isolated from healthy individuals and characterized as in our previous publications, there is no information on the number of donors, age, sex, tooth source, or whether the cells were pooled. Accordingly, as a primary cell culture, the results cannot be generalized to other PDLC populations. Another limitation is the distinct cellular origin of PDLCs and SCs. As such, PDLCs and SCs are presented and discussed as independent, complementary arms of the study rather than as a direct comparison, their results should be considered separately.

Finally, LPS+IFN-γ stimulation does not fully reflect the complexity of PD pathogenesis, as it represents only a transient pro-inflammatory microenvironment. Further characterization of the cellular response is needed over the long term, including protein-level validation and the examination of other cytokines.

## Conclusions

PDLCs exhibited a pro-inflammatory transcriptional response to LPS+IFN-γ, whereas SCs were unresponsive or showed downregulated expression of pro-inflammatory markers. Both cell lineages independently maintained their viability and wound-closure capacity. SCs formed increased mineralized matrix after transient inflammatory priming with LPS and IFN-γ. Further studies are needed to investigate additional cellular functions of PDLCs and SCs, including their immunoregulatory, anti-inflammatory, and osteoclastogenic capacities.

## Data Availability

The datasets generated during and/or analyzed during the current study are available from the corresponding author on reasonable request.
